# Cutaneous candidiasis caused by *Candida kefyr*

**DOI:** 10.11604/pamj.2021.38.178.28054

**Published:** 2021-02-16

**Authors:** Rosani Sri Camelia Nurdin, Sri Vitayani, Safruddin Amin, Dirmawati Kadir, Widyawati Djamaluddin, Anni Adriani

**Affiliations:** 1 Department of Dermatology and Venereology, Faculty of Medicine, Hasanuddin University, Makassar, South Sulawesi, Indonesia

**Keywords:** *Candida kefyr*, cutaneous candidiasis, fermentation test

## Abstract

Candidiasis is an acute or subacute fungal infection caused by fungi that belongs to candida genus, with Candida albicansbeing the most frequent causative agent. Candida kefyr is a rare cause of candidiasis which has been reported in systemic candidiasis and deep infections. However, to date, it has never been reported as a cause in dermatophytosis. We report a case of candidiasis caused by Candida kefyr in a 72-year-old woman with a chief complaint of pruritic erythematous rash on the back from one day prior to admission. Diagnosis was established based on clinical features, direct microscopic examination with 10% potassium hydroxide solution, gram staining. The fungal species was determined by carbohydrate fermentation test which showed a positive result for Candida kefyr. The patient was treated with miconazole cream and fusidic cream and showed significant clinical improvement.

## Introduction

Candidiasis is an acute or subacute fungal infection caused by the fungus of the genus candida, especially *Candida albicans*, and can affect the mouth, vagina, skin, nails, bronchi or the lungs, sometimes causing septicemia, endocarditis or meningitis [1]. The most common cause of cutaneous candidiasis is *Candida albicans*. A study showed that *Candida albicans* (48%) was the most often isolated species from the skin scrapings of cutaneous candidiasis patients, followed by *Candida kruzei* (16.1%), *Candida glabrata* (13.5%), *Candida kefyr* (7.4%), *Candida parapsillosis* (4.8%), *Candida tropicalis* (1.7%) and other candida species (8.5%). *Candida kefyr* is a rare cause of cutaneous candidiasis [2]. Candida infections occur if there are factors that nourish their growth or facilitate tissue invasion, such as weak host immune system. The causative factors consist of endogenous factors, such as physiological changes in pregnancy, obesity, iatrogenic, endocrinopathy, diabetes mellitus (DM), chronic disease, age and immunologic, and exogenous factors such as climate, skin cleanliness, foot bath habits and contact with those who suffer the same disease [3].

Candidiasis can be found throughout the world. The incidence of the disease is the same in men and women [1]. This disease can affect all ages increases in infants and elderly [1]. Cutaneous candidiasis is divided into two types namely intertriginous and granulomatous candidiasis. In intertriginous candidiasis, lesions occur on the skin folds of the armpits, groin, intergluteal, breast fold, between fingers or toes, glans penis, and umbilicus in the form of streaks that are demarcated, scaly, wet and erythematous [1]. Lesions are surrounded by dots in the form of small vesicles and pustules or bullae that leave erosive areas [1]. On the other hand, granulomatous candidiasis is more common in children and presents as reddish papules covered in thick brownish yellow crusts and are firmly attached to the base. This lesion may appear like a 2cm long horn and can often be found on the face, head, nails, body, and legs. We report a case of cutaneous candidiasis caused by *Candida kefyr* in a 72-year-old woman who responded well to topical antifungal therapy.

## Patient and observation

A 72-year-old woman was consulted to the Wahidin Sudirohusodo Hospital, Makassar, South Sulawesi, Indonesia, with a chief complaint of blackish red spots on the posterior trunk since one day before admission. The initial lesion was unknown. Complaints of itching is unknown due to patient´s decreased of consciousness state. The patient had a long history of immobilize for 14 days. History of similar complaint from the family was denied. History of diabetes mellitus was absent. Patient had a history of syphilis with HIV infection and got therapy of intramuscular injection 2.4 million unit of benzathine penicillin G for three consecutive weeks. He was also treated with antiretroviral therapy (ART) with tenofovir 300mg, lamivudine 300mg, and efavirenz 600mg.

**Figure 1:**
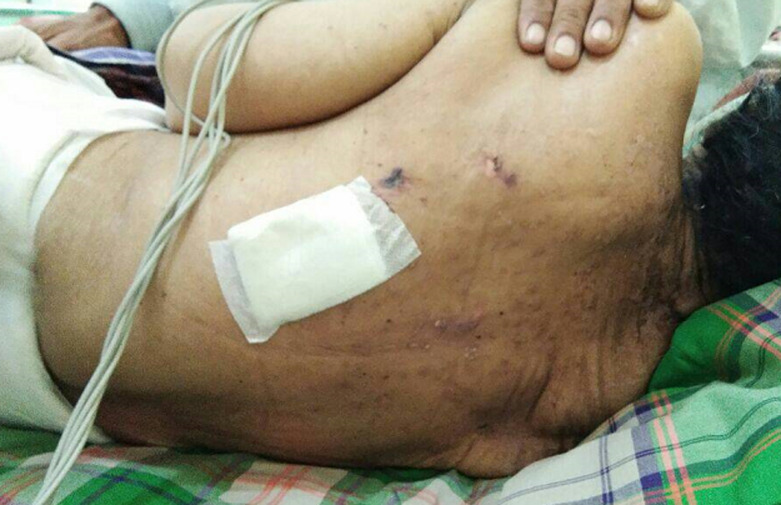
first day, hyperpigmentation macules accompanied by crusting, squama, and macular erythema in the posterior trunk area

**Figure 2:**
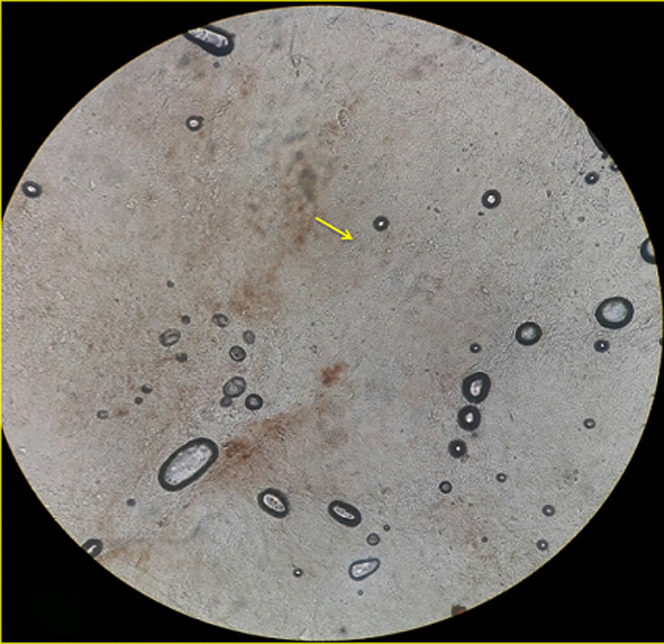
examination of 10% KOH showing clustered spores and pseudo hyphae

**Figure 3:**
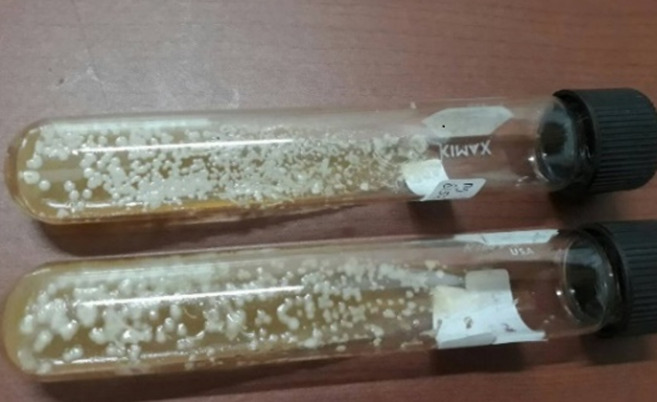
macroscopic culture in Sabouraud’s Dextrose Agar (SDA) media showing mucoid brownish white colonies with a smooth surface

**Figure 4:**
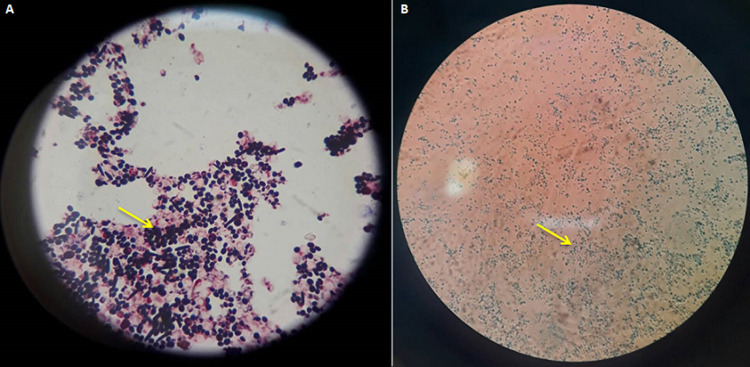
A) gram staining showed purple-colored fungal colony, yeast cells and budding spores; B) LCB (lactofenol cotton blue) staining showed positive results for candida

**Figure 5:**
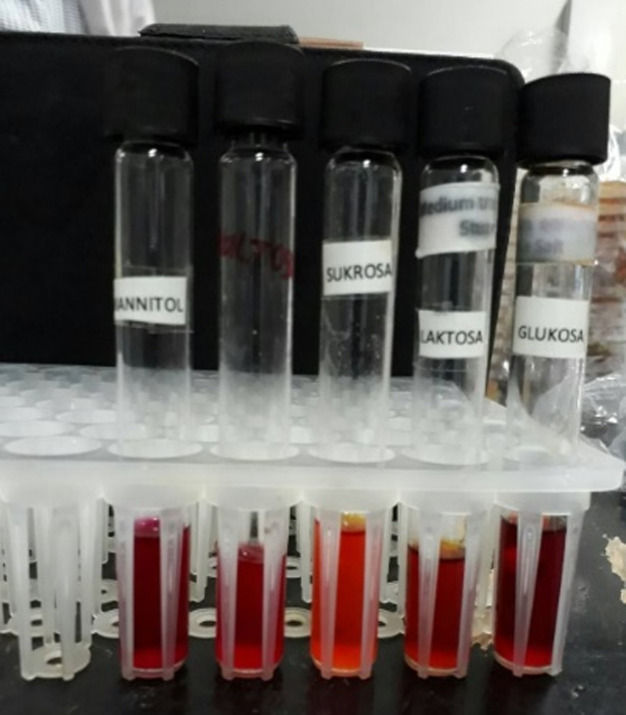
carbohydrate fermentation test resulted in a change in colour from purple to orange in glucose, sucrose, and lactose, and gas formation

Physical examination showed blood pressure of 100/80 mmHg, heart rate of 80 beats per minute, respiratory rate of 18 beats per minute, and temperature 36.5°C. Laboratory blood tests were within normal limits. Neurologic examination showed decreased awareness and lateralization of both sides. Dermatological examination of the posterior trunk region found hyperpigmented macules accompanied by crust, squama and erythematous macules (Figure 1). Based on the history taking and physical examination, the patient was diagnosed with sepsis, suspected meningoencephalitis, hypoalbuminemia, community-acquired pneumonia, and cutaneous candidiasis. The patient was given citicoline 500mg/12 hours/IV, omeprazole 40mg/12 hours/IV, ceftriaxone 2gr/12 hours/IV, vancomycin 1gr/12 hours/IV, vitamin B complex 1gr/24 hours/intravenously, acetazolamide 250mg/12 hours/nasogastric tube, and paracetamol 1gr/8 hours/intravenously. Topical fusidic acid cream was also applied on ulcerative lesions to avoid bacterial superinfection. For the cutaneous candidiasis, patient was treated with miconazole cream twice daily in the morning and evening and fusidic acid cream for 14 days. Direct microscopic examination using 10% potassium hydroxide (KOH) and culture were done to for further investigation on the causative agent. Direct microscopic of the skin scrapings on the back using 10% KOH showed clustered spores and pseudohyphae (Figure 2). Culture in Sabouraud Dextrose Agar (SDA) media added with chloramphenicol incubated at 25°-30°C showed mucoid brownish white colonies with smooth surfaces on the third day (Figure 3), typical of the fungus *Candida sp*. One week later, gram staining was carried out and positive purple color mildews were obtained (Figure 4). There were yeast cells and budding spores which were consistent with candida species. Further examination was carried out using carbohydrate fermentation to determine the type of candida which showed a change in color from purple to orange in glucose, sucrose, and lactose and gas formed in each of the reagents (Figure 5). The findings from carbohydrate fermentation test supported *Candida kefyr* as the causative agent (Table 1).

**Table 1:** identification of *Candida sp* types from carbohydrate fermentation tests

Species	Fermentation Process			
	G	M	S	L
*Candida albicans*	A+G	A+G*	A	-
*Candida tropicalis*	A-G	A-G	A+G	-
***Candida kefyr***	**A+G**	**-**	**A+G**	**A+G**
*Candida krusei*	A+G	-	(A)	-
*Candida parapsilosis*	A+G	(A)	-	-
*Candida guillermondii*	A+G	-	A+G	-
*Candida stellatoidea*	A+G	A+G	(A)	-

G: glucose; M: Maltose; S: sucrose; L: Lactose (A): can be acid or not; G *: sometimes there is no gas; A: acid; G: gas

**Figure 6:**
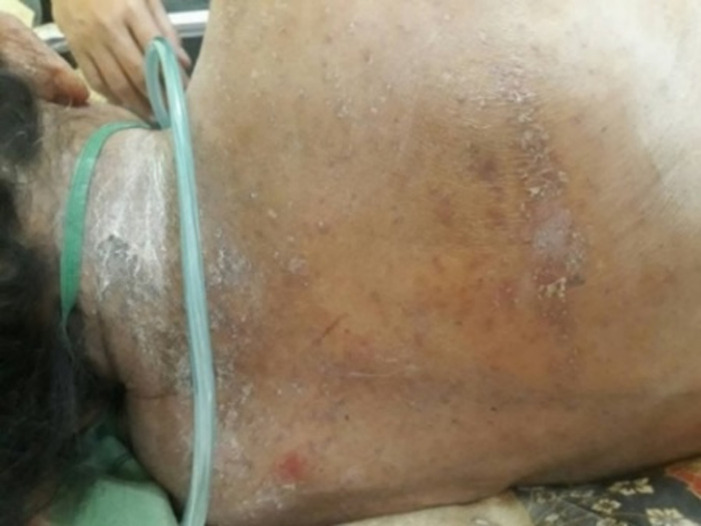
dermatologic examination on the fifth day showed the lesions on the posterior trunk had dried out

Data from the history taking, physical examination, and supporting examinations concluded a diagnosis of cutaneous candidiasis caused by *Candida kefyr*. On the fifth day of treatment the lesion showed marked improvement (Figure 6). Fusidic acid was dropped and only miconazole was continued. The patient was eventually discharged from the neurology department on the sixth day.

## Discussion

In this case the patient was diagnosed with cutaneous candidiasis based on history taking, physical examination, and supporting examinations. In this case, the patient is a 72-year-old woman. According to the literature, the incidence of the disease is the same in men and women. Risk of candidiasis increases in infants and elderly [1]. The diagnosis of candidiasis was made through history taking, physical examination and supporting investigations. On physical examination of the posterior trunk, hyperpigmented macules accompanied by crusting, scales, and erythematous macules were found. According to the literature, in vulvovaginal candidiasis caused by *Candida kefyr*, irritation and burning are more prominent than itching and is not accompanied by fluor albus. Clinically the main complaints are vaginal erythema, indifferent to that of *Candida albicans* [4].

Skin scrapings with 10% KOH solution is the easiest and most effective way to diagnose candidiasis but it cannot identify a specific etiology and is less sensitive than culture [1]. Positive skin scraping test for candidiasis show yeast cells, blastospores, or pseudohyphae [1,4,5]. In addition, specimens will also show purple color when examined using gram staining. Identification of fungi was done by culturing the specimens on SDA with added antibiotics (chloramphenicol) to prevent bacterial growth [6]. It is a sensitive examination to diagnose candida infections [1]. The culture is incubated at a temperature of 25°- 30°C. After 24-48 hours of culture, white colony was found, rounded convex with a distinctive odor of yeast, wet mucoid surface smooth and wrinkled [2,7]. Species identification can be done by germ tube test, chlamydospores assessment, assimilation and fermentation tests, and CHROM agar candida [8,9]. In this case we use carbohydrate fermentation test as this test is low-cost, simple, effective, sensitive, and specific [9]. This process requires carbohydrates as a source of carbon [9]. Kefyr species can assimilate and ferment glucose, sucrose, and lactose. A total of four tubes each containing glucose, maltose, sucrose, and lactose with added phenol red as an indicator were used. A change in color from red to yellow indicates the formation of acids [9,10]. To determine the formation of gas, durham cylinders that are placed upside down in a test tube were used. The gas formed will appear as an empty chamber in the Durham tube [9,10]. In *Candida kefyr*, fermentation can be observed on tubes containing glucose, sucrose, and lactose which is marked by color change and gas formation. In this case, the fermentation carbohydrate test showed positive results in tubes containing glucose, sucrose, and lactose as shown by color change and gas formation, confirming the presence of *Candida kefyr* [9,10].

## Conclusion

This case reports the first case of cutaneous candidiasis caused by *Candida kefyr*.
